# History, philosophy, and science education: reflections on genetics 20 years after the human genome project

**DOI:** 10.1007/s40656-023-00569-4

**Published:** 2023-04-25

**Authors:** Robert Meunier

**Affiliations:** Institute for History of Medicine and Science Studies, University of Lübeck, Königstrasse 42, 23552 Lübeck, Germany

Kampourakis, K. (2017). *Making Sense of Genes*. Cambridge University Press.

Rheinberger, H.-J., & Müller-Wille, S. (2017). *The Gene: From Genetics to Postgenomics*. University of Chicago Press.

They did it again! Kostas Kampourakis begins his book *Making sense of genes* with a few examples of headlines in respectable newspapers. Among them a 2014 headline from *The Guardian*: “‘Happy gene’ may increase chances of romantic relationships”. On October 20, 2021, the same outlet published a piece titled “Your green credentials may be linked to your genes, study says”. Most likely, there were many similar articles in-between.

The latter reports on a study that surveyed identical and non-identical twins on their concern for nature, environmental movement activism, and personal conservation behavior. The fact that identical twins had more similar responses than non-identical twins is interpreted by the researchers as “suggesting genetic influences on these phenotypes” (Chang et al., 2021, p. 3). This result is then connected to an evolutionary narrative about altruistic and cooperative behavior.

Of course, the researchers are quick to emphasize that such traits are caused by a “combination of genes and environments” and that this is “just about probability, not determinism.” And for sure, the journalist is critical. As is common for such reports, another expert, “who was not involved in the study,” is asked to evaluate the results. This researcher emphasizes that a large number of genes are involved in many processes, which contribute to such traits. He also points out that climate change, after all, is a “political problem”^1^.

No outrageous statements are made here. No one identifies a “gene for environmentalism.” The researchers use an established rhetoric to hedge their claims, and the journalist brings in critical peers. And yet, it appears that we haven’t come a long way since the days when the eugenicist Charles Davenport postulated genes “for” about everything, famously including *thalassophilia*. What will the reader of the *Guardian* piece come away with? Well, most likely that there are genes for environmentalism. And the outcome could not be more fatal in this case (literally!). Because ever since Francis Galton began to discuss human behavior in terms of heredity, what followed from such allocations of genetic responsibilities has been a deflection from the responsibility of a moral agent. If I do not feel inclined to act in a sustainable manner, then it is probably simply not in my nature.

There is so much amazing science out there that deserves more publicity. But if any third-rate scientific journal publishes a study that links genes to some interesting behavior, be sure some major news outlet picks it up. The fact that humans have different attitudes and show different behaviors never ceases to irritate people. Any claim that seems to explain these differences is welcome, no matter how vague or simplistic (because these are basically the two options available to make such claims).

For someone who knows the science studies literature on genetics (I am following Kampourakis here in using the term ‘genetics’ broadly to include genomic and post-genomic studies concerned with genes or genetic material), the continued prevalence of such reports is remarkable. And it is also the reason why Kostas Kampourakis’ book is so relevant even 20 years after the Human Genome Project (HGP) and the accompanying deluge of books and articles critical of gene centrism and genetic determinism (e.g., Kevles & Hood, 1993; Lewontin, 1993, 2000; Nelkin & Lindee, 1995; Keller, 2000; Sloan, 2000; Barnes & Dupré, 2008; see also Gannett, 2022).

Most people know fairly little about genetics but are nonetheless frequently confronted with claims about genes regarding crucial aspects of their life, from behavior to disease. Accordingly, for most people it is difficult to interpret such claims. Kampourakis’ book addresses anyone who wishes to gain competence in evaluating such information and in particular those who teach genetics in schools and universities, and to biology as well as humanities students. It would also be useful to physicians and health-care professionals as well as biologists who are attentive to the ways in which they communicate their scientific or diagnostic results, and to journalists disseminating research outputs. The book focuses on science education and communication, but it draws on arguments from the history and philosophy of genetics. The historical genealogies of contemporary discourse (Chs. 1–4) as well as the philosophical interpretation of the complexities of genetics (Chs. 9–12) are substantial and constitute an important framing for the discussion of how genes figure in public and professional discourse regarding biology and medicine, which is at the core of the book (Chs. 5–8). However, readers mainly interested in the history of genetics might want to consult the second book discussed below, while readers with a focus on philosophical issues might turn to Griffiths and Stotz’s *Genetics and Philosophy* (2013).

Kampourakis’ book is strongest where it explains those biological processes or relations that lie at the heart of the claims about the influence of genes on traits or diseases that lay persons encounter in various contexts from newspaper articles to the results of direct-to-consumer genetic tests. Chapter 7, for instance, uses concrete examples of ‘genetic’ diseases to explain the relation of genes, gene products, and environments to disease phenotypes. Also, the various technologies and approaches by which genetic evidence is produced are explained, for instance, genome-wide association studies (GWAS) in Chap. 6. Most laudably, notoriously difficult to understand concepts such as heritability (Ch. 10) are well explained and made intelligible. The same is true for the statistical nature of most claims about the relation between genes and diseases such as cancer; the meaning and adequate interpretation of such claims is clarified in Chap. 12.

Chapter 5, however, constitutes a conundrum, which points to an inherent problem of the book. The chapter is meant to show that the problem that the book addresses – the prevalence of genetic determinism in public and professional discourse – indeed exists. However, most of the studies cited, which look at messages from newspapers and movies and investigate the attitudes of lay people, students, and health-care professionals cannot really substantiate the claim. In fact, they provide either relatively weak evidence for the alleged problem or even evidence to the contrary. Already Condit (1999) seems to conclude that genetic determinism neither dominates public discourse on genetics, nor does it increase over the course of the twentieth century. It is very honest to present this evidence that does not fully support the main assumption of the book, as the author admits. At this point, the author goes on to suggest that the intuition of essentialism (underlying determinism) could be inborn (pp. 94–95), a view which would represent exactly the kind of deterministic claim that the book problematizes! All this remains unsatisfactory, especially if you *agree* with the author that the problem is real.

In my view, part of the problem is that the book operates with a caricature of genetic determinism (as well as genetic essentialism and reductionism). It begins by defining genetic determinism as the view that “genes invariably determine characters, so that the outcomes are just a little, or not at all, affected by changes in the environment” (p. 6). Accordingly, the author (and presumably also the researchers who conducted the cited studies on the prevalence of genetic determinism) interprets as non-deterministic any presentation or perception of genetic claims that acknowledges an interaction of genes and environment or the probabilistic nature of genetic influence etc. For this reason, the only thing these studies can show is that people are not entirely naïve about genetics (i.e., they do not hold the strongest version of determinism as portrayed in the caricature). While this result is reassuring, the point is that even if one gets the basics right (genes only account for variation; genes interact with the environment; genes exert their influence in a probabilistic manner, etc.), one can still get many things wrong. And indeed, the book in other parts does an excellent job in showing just that!

Also the notion of “genes for x”, where x would typically be some morphological or behavioral character or disease, is interpreted in the maximally uncharitable manner by Kampourakis when he debunks it by stating that “genes do not alone produce characters or disease” (p. 8, p. 155, p. 170; note the shift from “determine” to “produce”) or characterizes the notion as referring to “genes as character makers” (p. 32, p. 188; in the latter location the phrase is meant to characterize a quotation which, however, could well be read as depicting genes as “character-difference makers” instead). It would of course be hard to prove, but I would argue that no geneticist in the twentieth century held a view that could be fairly represented by such formulations (i.e., that genes alone ‘produce’ or ‘make’ characters). In fact, not even the organically growing hereditary particles postulated in the speculative theories of the nineteenth century (from Darwin’s “gemmules” to Weismann’s “biophores” and “determinants”, see e.g. Churchill, 2015) can be interpreted as character makers. First, because the concept of character always implied a difference (it stems from taxonomy), while what grows is a part; and second, because these particles were mainly meant to explain the presence of various cell types rather than characters which would typically involve (differences in) many cell types. The result of this exaggeration of the determinism that is seemingly implied in common talk about genes, is that the more subtle, but also more common and thus more problematic mis-conceptions are sidelined. In other words, the book could have been stronger if it had acknowledged that views of genes as difference makers, as interacting with the environment, and as acting probabilistically are widely held and that mis-representations and mis-interpretations arise despite this common wisdom.

Unlike the second book discussed in this review, the focus of Kampourakis’ book is not on the science of genetics, but on the way it is communicated and understood by practitioners or lay-audiences. Nonetheless, there are many places where Kampourakis seems to suggest that also genetic research itself is permeated by a problematic understanding of genes. For example, Ch. 9 gives the impression that the inadequate metaphor of the genome as a ‘blueprint’ for an organism constitutes a widely held view in biology. I doubt that this is the case. It has, of course, often been shown by science studies scholars that scientists are metaphysically or ideologically biased or use misleading metaphors. But then, again, the target of criticism here, at least regarding the role of genes in development, behavior, and disease, often seems to be a strawman. When philosophers or other commentators of the life sciences debunk a deterministic ‘received view’ in genetics, they typically marshal a host of genetic mechanisms that illustrate the complexity of genetic processes, from alternative splicing to RNA editing to epigenetics. These insights, however, come from the science of genetics. One might argue that they were – at least for a considerable amount of time – marginalized. There are indeed reasons for conservatism in science (see e.g., Bedessem, 2021) and, in any case, new ideas have to be scrutinized by several communities before they become generally accepted. Nonetheless, there are also incentives to pick up new findings that complicate the received picture, as they open up new avenues for original research and publications. Accordingly, those actually concerned with the role of genes in development, usually do not hold a simplistic picture of gene action. Not rarely the quotes taken to represent the naïve and allegedly mainstream view are either quite old, found in the literature belonging to a biological discipline not directly concerned with development or disease genetics (e.g., in population genetics texts), or taken from public-facing promissory statements. It is important therefore, when criticizing scientific views on the basis of other scientific findings, to be very explicit and precise about who holds a criticized view or neglects an alternative view and when exactly. The second book discussed in this essay seems to mainly resist the temptation to bash genetics as overly deterministic, reductionist, or as negligent of a variety of processes, by showing that the gene as well as the gene concept function as tools in illuminating the very complexities that appear to undermine the notion of the gene.

Hans-Jörg Rheinberger and Staffan Müller-Wille in their slim volume *The Gene: From Genetics to Postgenomics* keep their focus firmly on the gene in biological research. The broader social and cultural context is largely excluded. One could see this as a disadvantage of the book, especially in light of Kampourakis’ emphasis on the impact of genetics on public discourse. However, first, the book would not have been as short as it is had the authors included these dimensions, and its compactness is a true advantage, for instance, when using the book as teaching resource. Second, the authors have already presented a previous account that highlights the cultural context in which hereditary thinking developed and which it influenced in turn (Müller-Wille & Rheinberger, 2012). In contrast to this earlier book, which begins its narrative in the early modern period, the book discussed here deals primarily with the period from the late nineteenth to the twenty-first century and focusses – as the title suggests – on the gene, rather than on hereditary thinking broadly conceived. Unlike Kampourakis’ book, which addresses a broad audience interested in understanding genetic claims, this book mainly targets a scholarly audience. Given its introductory character, however, it will be suitable for teaching purposes in a variety of disciplines concerned with genetics either from a reflective or a practical perspective.

One might ask if such a book is needed, given that there are already many histories of genetics and the gene concept. The book is far from being redundant, however. One obvious reason is that the life sciences are moving at fast pace. Early histories, such as Carlson’s *The Gene: A Conceptual History* (1966) can still be informative about the early decades of genetic research and later accounts such as Keller’s *The Century of the Gene* (2000) can still stand as reflections about how genetics became molecular. However, each of these are influenced by the concerns at the time of writing and aged in particular ways, and, most of all, they do not contain accounts of developments in the twenty-first century. There have been many individual studies looking specifically at one or the other recent development in the life sciences, such as epigenetics, metagenomics, or systems and synthetic biology. But at this point, there are few works which discuss these developments in the context of a long-term history of genetics and the gene; Rheinberger and Müller-Wille discuss the latest developments in Chap. 9. The only contender that comes to mind is Michel Morange’s recent book *The Black Box of Biology* (2020), which constitutes an update and extension of his earlier *History of Molecular Biology* (1998); this book features whole chapters on post-genomic themes, but it is also much longer.

For sure, compacting a long and multidimensional history of a prominent concept such as the gene does not come without choices, omissions, and synopsis. Rheinberger and Müller-Wille’s book only highlights turning points in the trajectory of the concept; but for readers who need to zoom in on a particular historical episode, it provides references to the relevant literature. The text offers more, however, than a mere overview in the sense of displaying key developments in biology diachronically and synchronically. It develops a narrative that highlights historical as much as systematic connections between events. Sometimes these connections are more of pedagogical value rather than representing a deeper historical pattern. Such is the case when the development of classical genetics and molecular genetics are narrated as analogous movements from simple to complex models and methods (Chs. 3–6) or when the impact of genetics on evolutionary biology on the one hand and developmental biology on the other hand are implicitly parallelized (Chs. 5 and 8). However, the most important and insightful notion emerging from the narrative is that genes have such a central position in biology not necessarily for ontological reasons, but for heuristic or pragmatic reasons. DNA elements can be used as instruments for manipulation to study a vast variety of processes and mechanisms from development to diseases and from epigenetics to molecular interaction network dynamics. Hence, also the concept of the gene is a starting point for planning experiments and interpreting results.

The authors begin with the insight that – despite all the complications of cellular biology and their consequences for heredity, development, and evolution seemingly undermining the gene concept – reference to genes remains omnipresent and central not only outside science, but also at the cutting-edge of research in the life sciences (Ch. 1). A key to making sense of this paradoxical situation is provided in Chap. 7, where the focus is on the development of molecular technologies. Already before, but certainly after the introduction of recombinant DNA technologies and other sophisticated devices of the molecular toolbox, genes were not only *epistemic* objects that continuously changed regarding the properties and relations ascribed to them when new analytic technologies were applied to them, but they became *technical* objects themselves that were used to study other objects or processes (see Rheinberger, 1997 on epistemic vs technical objects). The focus on research technologies then also ties the latest, post-genomic developments into the picture, e.g., regarding the impact of microarrays and next-generation sequencing (Ch. 9). Throughout the book, the authors emphasize the context of experimental systems from early hybridization experiments to recent high-throughput technologies to show how genes – as concepts and as objects singled out by the concept at given times or in specific situations – functioned to elucidate processes that undermined deterministic or essentialist interpretations of genetics, from the role of environmental factors to the system properties of cells.

It is this insight into the epistemic and pragmatic roles of genes in biological research (discussed also by others, e.g., Waters, 2007 or Gannett, 1999) that enables a view that departs from the usual bashing of gene-centrism. One is tempted to employ the notorious pendulum metaphor here. Where earlier histories on the occasion of the Mendel centennial and in the wake of the elucidation of the structure of DNA and the genetic code tended to be caught up in heroic and progressive success narratives of science, histories written in response to overblown claims in the context of the HGP tended to be overly critical, often vigorously attacking the same reductionist/determinist strawman that still haunts Kampourakis’ book in certain places. But rather than locating the account by Rheinberger and Müller-Wille in a sober middle position, it seems more appropriate to say that these authors have their own specific perspective that is equally located in its time. As scholars who contributed strongly to the practice turn in the history and philosophy of science, their account is informed by the way the gene as a concept but also as an epistemic and a technical object functions in the pragmatics of research. That is to say, rather than focusing on what researchers at a given time said about what genes are or do, they focus on how this understanding is mediated by the ways in which researchers at a given time were able to interact with genetic materials, use these interactions to find out yet other things about these materials or about other objects or processes, or control processes in agricultural or medical contexts. Likewise, the concept of the gene is a tool to represent and communicate this practical knowledge across disciplinary boundaries rather than a term used to express immutable truths about life.

If there is something missing in the book, one might say that the authors missed the chance of historicizing the philosophical and historiographic discourses about the gene. This then is a task that is still open for others to pursue. In fact, a special issue from 2022 focusing on Rheinberger’s work contains many interesting perspectives on the recent history of science studies scholarship concerning the life sciences (Keuck & Nickelsen, 2022).

When I spilled some ink on certain shortcomings of Kampourakis’ book, then that was only because they moderately diminish the value of an otherwise extremely relevant and well-written book. Looking at the two books discussed here together with Griffiths and Stotz’s *Philosophy and Genetics* and Morange’s recently updated detailed history, it is fair to say that the last ten years have equipped us with a high-quality library of books for a critical and historically informed understanding of genetic discourse, which is suitable for designing curricula of HPS courses as much as in science education, as well as serving as a starting point for further scholarly discussions of related topics (for a more compressed overview, see also Meunier, 2022).

## Footnotes

1 https://www.theguardian.com/environment/2021/oct/20/your-green-credentials-may-be-linked-to-your-genes-scientists-say (Accessed October 1, 2022)
